# The Heterochromatic Barrier to DNA Double Strand Break Repair: How to Get the Entry Visa

**DOI:** 10.3390/ijms130911844

**Published:** 2012-09-19

**Authors:** Aaron A. Goodarzi, Penny A. Jeggo

**Affiliations:** 1 Southern Alberta Cancer Research Institute, Departments of Biochemistry & Molecular Biology and Oncology, University of Calgary, Calgary, Alberta, T2N 4N1, Canada; 2 Genome Damage and Stability Centre, University of Sussex, Brighton, BN1 9RQ, United Kingdom

**Keywords:** DNA non-homologous end-joining, chromatin, heterochromatin, damage response signaling, ataxia telangiectasia mutated, homologous recombination

## Abstract

Over recent decades, a deep understanding of pathways that repair DNA double strand breaks (DSB) has been gained from biochemical, structural, biophysical and cellular studies. DNA non-homologous end-joining (NHEJ) and homologous recombination (HR) represent the two major DSB repair pathways, and both processes are now well understood. Recent work has demonstrated that the chromatin environment at a DSB significantly impacts upon DSB repair and that, moreover, dramatic modifications arise in the chromatin surrounding a DSB. Chromatin is broadly divided into open, transcriptionally active, euchromatin (EC) and highly compacted, transcriptionally inert, heterochromatin (HC), although these represent extremes of a spectrum. The HC superstructure restricts both DSB repair and damage response signaling. Moreover, DSBs within HC (HC-DSBs) are rapidly relocalized to the EC-HC interface. The damage response protein kinase, ataxia telangiectasia mutated (ATM), is required for HC-DSB repair but is dispensable for the relocalization of HC-DSBs. It has been proposed that ATM signaling enhances HC relaxation in the DSB vicinity and that this is a prerequisite for HC-DSB repair. Hence, ATM is essential for repair of HC-DSBs. Here, we discuss how HC impacts upon the response to DSBs and how ATM overcomes the barrier that HC poses to repair.

## 1. Introduction

A broad range of studies including biochemical, structural and biophysical approaches have provided substantial detail and insight into the two most significant pathways of DNA double strand break (DSB) repair, namely DNA non-homologous end-joining (NHEJ) and homologous recombination (HR). Cell biology approaches have revealed that *in vivo* NHEJ functions throughout the cell cycle while HR is restricted to late S/G2 phase. *In vivo* approaches have also demonstrated that the operation of these pathways and the choice between them, which appears to be determined by DNA end resection, is highly influenced by the chromatin environment surrounding the DSB. Of relevance to this review, the response to DSBs located within regions of heterochromatin (HC-DSBs) is substantially distinct to that which arises at DSBs formed in euchromatin (EC-DSBs). Additionally, there is increasing evidence that a significant role of the DNA damage response kinase, ataxia telangiectasia mutated (ATM), is to modify the chromatin environment in the DSB vicinity. Whilst these modifications can include changes in phosphorylation, ubiquitylation, SUMOylation and methylation, many of which enhance chromatin relaxation, there is, paradoxically, evidence that proteins promoting chromatin compaction, such as KAP-1, HP1 and HDACs, can become relocalized and/or recruited to DSBs [1–4]. Thus, depending on the chromatin environment surrounding the DSB and a range of additional factors, it appears that chromatin can either undergo relaxation or compaction. Such changes can serve to modulate the DSB repair process and very likely serve to preclude translocations or inhibit transactions, such as transcription, that may interfere with the repair process. Further, these changes can be transient or sustained throughout, and even beyond, the duration of repair. Whilst our understanding of the mechanisms underlying many of these chromatin changes and the rationale for them remains in their infancy, the changes that take place at HC-DSBs have a marked impact on DSB repair and damage response signaling. The focus, here, is to review these changes and to consider how they influence the DNA damage response (DDR) and the choice of DSB repair pathway.

## 2. Overview of the DNA Damage Response and Chromatin Changes that Arise at DNA Double Strand Breaks

The DDR to DSB formation encompasses repair pathways and a signal transduction response, which causes significant changes to the chromatin environment at a DSB. DNA non-homologous end-joining (NHEJ), which involves the Ku70-Ku80 heterodimer, the DNA dependent protein kinase catalytic subunit (DNA-PKcs), XRCC4, DNA ligase IV and XLF, represents the major DSB repair pathway in mammalian cells [5,6]. Homologous recombination (HR) also functions in late S-G2 phase cells, when a sister homologue is available as an undamaged template [5]. ATM lies at the heart of the major signaling response to DSBs although the related kinase, ATR, can play a role in S and G2 phase cells [7]. Interestingly, NHEJ functions predominantly independently of ATM-dependent signaling and vice-versa, ATM signaling is not dependent upon DSB repair. The MRE11-RAD50-NBS1 (MRN) complex serves as the initial DSB sensor, both recruiting and activating ATM. ATM and DNA-PK can redundantly phosphorylate H2AX, the first step in the signaling process, generating γH2AX [8]. Subsequent changes include the recruitment of the mediator protein, MDC1, the ubiquitin ligases, RNF8 and RNF168, which effect histone ubiquitylation and regulate exposure of methylated groups, which in turn promotes recruitment of another mediator protein, 53BP1 [9]. RNF8 and RNF168 predominantly ubiquitylate histones H2A and H2AX [10,11]; histone H2B can also undergo ubiquitylation by the RNF20-RNF40 heterodimer [12,13]. In addition, SUMOylation of histones by PIAS1 and PIAS4 takes place, promoting recruitment of the SUMO-targeted ubiquitin ligase RNF4 [14–16]. Collectively, this co-ordinated assembly process leads to irradiation induced foci formation (IRIF), which can expand up to megabase pair regions around the DSB. In addition to these changes which promote IRIF formation, a number of additional events have been reported, which can result in transient changes in histone acetylation and/or methylation. We aim here to discuss the response to DSBs that arise within regions of HC. We recommend several good reviews for a more generalized review of the enormous chromatin changes that are affected by the DDR [9,17–20].

## 3. Evidence that Heterochromatin Influences the Signal Transduction Response at a DSB

A range of studies have demonstrated that chromatin undergoes rapid decondensation at DSBs, leading to a more open configuration [21–23]. However, whilst important in showing that chromatin relaxation occurs at DSBs, these studies do not differentiate between events arising at highly compact HC from more open EC regions. One of the first studies demonstrating that HC is refractory to γH2AX modification was made in budding yeast, where γH2AX spreading from an endonuclease-induced DSB into the silent *HML* and *HMR* loci, which are largely deacetylated and heterochromatic, does not occur in contrast to efficient spreading into euchromatic sequences [24]. Supportive work in mammalian cells showed that γH2AX foci size diminishes with proximity to pericentric heterochromatin and can be enhanced by treatment with a histone deacetylase inhibitor [24]. A further study substantiated this notion using ChIP assays to detect γH2AX and found that γH2AX expansion was reduced in heterochromatic sequences [25]. Related, but somewhat distinct, is the observation that γH2AX foci are unevenly distributed early after IR exposure and arise preferentially in EC regions of the genome [26]. Thus, whilst γH2AX foci within EC can be observed within a few minutes, they do not form within densely DAPI-staining regions which are considered to represent compacted HC, but instead expand at slightly later times on the periphery of DAPI-dense regions [27,28]. A more recent study using high linear energy transfer (LET) radiation obtained following ion particle traversal, which generates DSBs in the particle tracks, showed that although the particles traverse cells in a straight trajectory, the path of subsequent γH2AX foci formation does not form in a linear manner but, rather, deviates and bends around the DAPI-dense or H3K9me3-rich domains, consistent with the relocalization of DSBs to the periphery of HC regions [29]. Finally, a study in Drosophila, where DAPI-bright heterochromatin containing highly repetitive sequences comprises approximately 30% of the genome, showed that γH2AX and RPA foci can be observed within HC regions at very early times but rapidly relocalize to the periphery and that RAD51 foci form only at the periphery [30]. Furthermore, the study showed that HC rapidly expands and becomes dynamic after radiation exposure. Although somewhat distinct to the situation in mammalian cells, there is striking overlap. Collectively, these findings demonstrate two important aspects of DDR signaling; firstly, they provide strong evidence that modifications to the chromatin that arise during the DDR are inhibited by the HC superstructure and secondly, they demonstrate that DSBs formed within HC regions migrate to the periphery of such regions where DDR modifications ensue. This raises the important issue of whether a failure to observe IRIF within HC regions is, in fact, due to a barrier posed by the HC or whether it is simply explained by the migration of DNA regions with DSBs to the HC periphery. Thus, formally it is possible that HC is not intrinsically restrictive to histone modifications but rather that the regions of HC with DSBs become relocalized to HC-EC interface regions.

### 3.1. Movement of DSB-Containing DNA to the Periphery of Chromocentres

The use of carbon ion trajectory irradiation has shown convincingly that DSB-containing regions of DNA migrate to the periphery of HC regions [29]. In Drosophila it was suggested that this relocalization requires cell cycle checkpoint proteins and is not observed in cells lacking the SMC5-SMC6 complex [30]. However, SMC5-SMC6 is required for normal heterochromatinization. Consequently, it is possible that DSB movement to the HC periphery does not occur in cells lacking SMC5-SMC6 simply because the HC is insufficiently compacted. In our own studies using mammalian cells, we have not observed any genetic deficiencies that prevent this DSB relocalization and the process certainly does not require DDR checkpoint kinases [27,28]. We, therefore, favor a model whereby the presence of a DSB itself causes relaxation in chromatin compaction and that the HC superstructure is dynamically changing and influenced by the presence of DSBs. Thus, we suggest that the rapid movement of DSBs to the periphery of HC regions represents a passive process that arises due to HC relaxation in the vicinity of a DSB. Consequently, the expansion of IRIF on the periphery of DAPI-rich regions is predominantly because that is where DSBs become located. Further complicating the interpretation of some of these studies is the possibility that highly compacted domains might render DNA less susceptible to DSB formation after IR. Consistent with this possibility, studies have shown that transcriptionally inert, condensed chromatin is less susceptible to DSB induction than expressed, decondensed domains. Nonetheless, in these studies, open chromatin did allow more efficient DSB repair [31,32].

### 3.2. HC Is also a Barrier to IRIF Expansion

**Figure 1:**
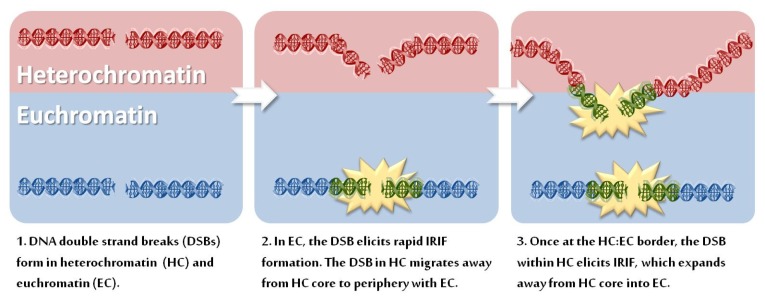
Differential irradiation induced foci (IRIF) formation between Euchromatin and Heterochromatin. (**1**) DNA double strand breaks (DSBs) form within either heterochromatin (red) or euchromatin (blue); (**2**) γH2AX occurs on chromatin at the DSB site (green) enabling the formation of the larger IRIF (yellow star) comprised of proteins such as Mre11, Rad50, NBS1, MDC1, RNF8, RNF168 and 53BP1. In heterochromatin, however, IRIF fail to form to a similar extent (or at all) at the same time point. Rather, the heterochromatic DSB relocates from the heterochromatic core to the peripheral zone bordering on euchromatin; (**3**) Once relocated to the heterochromatin:euchromatin border, the heterochromatic DSB elicits IRIF formation which expands into the surrounding euchromatic space.

Whilst some observations described above may be attributed to DSB movement within HC, there is distinct evidence that HC is refractory to IRIF expansion; for example, in the studies of Kim *et al.*, γH2AX modifications originating from an HO-induced DSB penetrate avidly into EC but not HC [24]. Further, a distinct approach has shown enhanced expansion of γH2AX foci at DSBs within HC relaxed by inhibition, depletion or mutation of defined HC proteins [26,33]. For example, greater γH2AX expansion is observed following addition of HDAC chemical inhibitors [26,34]. Such a consequence may not be unique to HC proteins, however, since haploinsufficiency of histone H1 has similarly been reported to confer an increase in the magnitude of DDR signaling, observed as enhanced phosphorylation of ATM substrates [35]. However, of more relevance in the present context, is the finding that depletion of the KRAB-associated protein 1 (KAP-1) co-repressor, a critical HC building factor, similarly led to enlarged IRIF. Moreover, a number of human disorders with disordered HC have been described, including immunodeficiency, centromeric region, facial anomalies syndrome type A (ICFa, caused by mutations in the DNA methyltransferase, DNMT3B), Hutchinson-Guildford Progeria syndrome (HGPS, caused by mutations in the nuclear envelope protein lamin A) and Rett syndrome (caused by mutations in a methyl-(CpG) DNA binding protein, MeCP2). Interestingly, cells derived from such patients also show larger γH2AX foci compared to those observed in control cell lines [33]. Moreover, the larger foci are predominantly due to enhanced encroachment of γH2AX foci into the dense DAPI-staining regions. Collectively, a broad range of studies have provided substantial evidence that HC is a barrier to the expansion of γH2AX spreading, although as a separate and potentially confusing issue, DSBs also relocate to the interface between HC and EC regions (depicted in Figure 1).

### 3.3. Can IRIF Formation Be Initiated in HC Regions?

In our own studies with DSBs induced by gamma or X-rays, we have not, even at early times post IR, detected γH2AX foci within the core of densely DAPI-staining regions. Indeed, we have observed differential kinetics of γH2AX foci formation at EC versus HC regions, consistent with the notion that IRIF predominately form on the periphery of HC [27,33]. However, further studies involving 3D imaging at early times post IR are required to verify whether IRIF can be observed in the HC interior at a time when they are first visible in EC regions. In contrast to these studies with X-ray induced DSBs, small IRIF have been observed to form within HC domains following exposure to carbon ion irradiation [29]. Whilst this might suggest that some level of IRIF formation can occur within HC, it is also possible that the substantially greater density and/or complexity of DNA strand breaks arising after high LET irradiation confers sufficient HC relaxation to allow some IRIF formation which may not occur at the less dense and/or complex DSBs induced by X-rays. Another interesting consideration is that “simple” (less dense, easily-ligatable) DSBs may escape detection in HC if they are repaired during the process of relocalization and IRIF formation in contrast to EC-DSBs where the more rapid IRIF formation may allow their detection [36]. As mentioned above, it has been reported in Drosophila that both γH2AX and RPA foci formation can occur within HC regions but that the loading of RAD51 is precluded [30]. Interestingly, in mammalian cells, we have observed that HC poses a greater barrier to RAD51 loading than to DSB end resection, suggesting that at least some DDR signaling can occur within HC regions (data not shown).

## 4. HC Is a Barrier to DSB Repair and Is Relieved by ATM-Dependent Signaling to KAP-1

While there remains some uncertainty about the relationship between DDR signaling and HC, there is strong evidence that HC poses a barrier to DSB repair. Most strikingly, the loss of ATM protein kinase activity confers a DSB repair defect and the persisting DSBs localize to the HC periphery, suggesting that ATM signaling is necessary to facilitate repair of HC-DSBs [27]. This is consistent with, although somewhat distinct to, the findings in Drosophila that HC represents a barrier to RAD51 deposition on resected DNA [30]. Interestingly, it appears that ATR rather than ATM functions in Drosophila to promote DSB repair in HC regions.

In addition to the requirement for ATM for HC-DSB repair, there is also a requirement for downstream components of ATM signaling including γH2AX, MRE11, NBS1, MDC1, RNF8, RNF168 and 53BP1 [28,37]. In contrast, EC-DSBs are repaired efficiently in the absence of any of these proteins. Further, knockdown of a range of distinct HC component proteins, including HP1 (α + β + γ), KAP-1 or HDAC1 + HDAC2, permits HC-DSB repair in the absence of ATM [27]. Additionally, mutation of HC proteins in the human syndromes previously discussed (ICFa, HGPS or Rett Syndrome) diminishes the requirement for ATM for HC-DSB repair. Collectively, these findings are consistent with the notion that the HC superstructure confers a barrier to DSB repair, which can be relieved by loss of any one of a number of proteins that contribute to the HC superstructure. Significantly, KAP-1 is an ATM substrate with an ATM-dependent phosphorylation site (S824) in its *C*-terminal region [38,39]. The fact that KAP-1 depletion by siRNA relieves the requirement for ATM for DSB repair does not necessitate that ATM functions via phosphorylating KAP-1, since loss of several HC components confers the same phenotype. However, expression of non-phosphorylatable S824A KAP-1 confers an HC-DSB repair defect independently of ATM status whilst expression of the phosphomimic S824D-KAP-1 alleviates the need for ATM for HC-DSB repair [27]. These findings, therefore, strongly argue that ATM’s major role in mediating HC-DSB repair is its ability to phosphorylate KAP-1 at S824. It should be noted that an additional phosphorylation site on KAP-1 has recently been identified (S473), but this does not represent a direct target of ATM/ATR [40,41]. Rather, KAP-1 S473 phosphorylation is mediated by Chk1 or Chk2 and, although contributing to the DDR (see below), it does not directly influence ATM-dependent DSB repair [40,42,43].

## 5. Fast and Slow DSB Repair Processes

In addition to distinct genetic requirements for HC versus EC-DSB repair, the repair of these two classes of DSBs occurs with markedly different kinetics [27,41]. For many years, it has been recognized that there are fast and slow components to DSB repair and recent studies have shown that the majority of HC-DSBs are repaired with slow kinetics via an ATM-dependent process (as discussed above). The basis underlying the slow kinetics, however, is unclear. One explanation is that the barrier posed by HC restricts the DSB repair process, conferring slow kinetics. However, siRNA-mediated depletion of KAP-1 (or other HC components) relieves the requirement for ATM for HC-DSB repair but does not impact upon the (slow) kinetics by which HC-DSBs are repaired. Significantly, the endonuclease, Artemis, is also required for the slow DSB repair process but depletion of KAP-1 does not overcome this requirement [44]. This raises the interesting possibility that the slow DSB repair process represents a somewhat distinct DNA end-joining mechanism to the fast process. Significantly, the slow DSB repair component requires core NHEJ proteins (DNA ligase IV and DNA-PKcs) in G1 phase but, in contrast, requires core HR factors (BRCA2, Rad51, Rad54) in G2 phase [37,45]. This striking result demonstrates an important impact of HC in determining the DSB repair pathway choice in G2 phase [46]. Interestingly, whereas ~15% of X-ray induced DSBs are repaired with slow kinetics, following exposure to high LET radiation (carbon ion particles), the majority (>80%) of DSBs are slowly repaired in G1 and G2 phase. Since the slow DSB repair process in G2 phase represents HR, the findings reveal that whereas only 15%–20% of X-ray induced DSBs are repaired by HR in G2, HR makes a much greater contribution to the repair of DSBs induced in G2 phases by high LET radiation [46]. Thus, the model we favor based on these findings is that core NHEJ makes an initial attempt to repair all DSBs in either G1 or G2 by a process that does not require endonuclease or exonuclease activity [46]. This process represents canonical NHEJ (c-NHEJ) and occurs with rapid kinetics. However, at least two factors may inhibit rapid repair by c-NHEJ; the heterochromatic superstructure and the complexity of DSB ends. If c-NHEJ cannot proceed rapidly, then we suggest that a slower process that likely involves resection and Artemis nuclease activity then ensues. In G1 phase, rejoining still occurs by a process that exploits c-NHEJ proteins, potentially representing a form of microhomology-mediated NHEJ, whilst in G2 phase, more extensive resection occurs and rejoining ensues by HR [46]. Neither of these processes represents alternative (Alt)-NHEJ, since the processes require DNA ligase IV and are unaffected by PARP inhibition (data not shown). Thus, we suggest that the slow kinetics is a consequence of end processing that takes place at these DSBs rather than being a direct consequence of the HC superstructure. However, there is clearly a complex set of factors that influence the speed of repair and the precise basis underlying the kinetics remains unclear.

## 6. Role of the Mediator Protein, 53BP1, for pKAP-1 Foci Formation and HC-DSB Repair

As stated above, 53BP1 shows a similar phenotype to ATM for DSB repair in G0/G1 phase—*i.e.*, it is required for the repair of HC-DSBs and its requirement can be overcome by siRNA-mediated knockdown of a number of HC component proteins [28]. 53BP1 recruitment is a terminal step in IRIF assembly and depends upon multiple upstream steps including the ubiquitylation of H2A/H2AX by RNF8-RNF168 and the degradation of KDM4A/JMJD2A to expose H4K20me2 [47,48]. Importantly, 53BP1 recruitment enhances the tethering of phosphorylated ATM at DSBs via a direct interaction between 53BP1 and the RAD50 component of the MRN complex [28,49]. Thus, the proposed model is that ATM is retained at DSB sites via a direct interaction with the NBS1 component of the MRN complex. MRN, itself, is partially tethered at IRIF via interactions with MDC1, which is itself recruited by γH2AX. However, a further critical step leading to ATM IRIF, particularly at later times post IR, relies on an interaction between the RAD50 component of MRN and 53BP1. Thus, 53BP1 is required for efficient ATM retention at the DSB site, although it is dispensable for ATM activation and its initial recruitment [28,48].

Two distinct modes of KAP-1 S824 phosphorylation (pKAP-1) have been delineated, the basis of which may derive from the different mechanisms of ATM retention [28,38,41]. At early times post IR, pKAP-1 forms in a pan-nuclear manner via an ATM-dependent but 53BP1-independent process. Whilst this could arise from activated ATM being dispersed in a pan-nuclear manner, there is evidence that KAP-1 is phosphorylated at DSB sites and becomes dispersed throughout the nucleus [38]. Nonetheless, pKAP-1 is or becomes chromatin bound, giving rise to pan nuclear, low intensity phosphorylated KAP-1. In parallel, pKAP-1 foci also arise, which effectively represent high density KAP-1 S824 phosphorylation at the DSB site [28]. Significantly pKAP-1 foci only form at HC-DSBs, classified by their localization at dense DAPI-staining, KAP-1-rich regions. Although it is difficult to visualize pKAP-1 foci at early times post IR exposure due to the high background of pan nuclear pKAP1, evidence for distinct foci was achieved by computer-assisted microscopy analysis of pKAP-1 *versus* KAP-1 signal above the pan-nuclear background staining. In this way, pKAP-1 foci were shown to be 53BP1-dependent (in contrast to pan nuclear pKAP-1), to form at only a subset of DSBs (HC-DSBs) and for the loss of these foci (taken to represent DSB repair) to be both 53BP1 and ATM-dependent. Thus, the evidence strongly suggests that HC-DSB repair requires pKAP-1 foci formation, whilst pan-nuclear pKAP-1 is not sufficient to promote HC-DSB repair.

A somewhat distinct finding is that KAP-1 as well as all three isoforms of HP1 are rapidly and transiently recruited to DSBs induced by laser microirradiation. KAP-1 and HP1α recruitment occurs via a p150CAF-1-dependent process whilst HP1α recruitment is KAP-1 dependent [1]. Thus, it was proposed that the retention of HP1α at damage sites may be linked to ATM-dependent phosphorylation of KAP-1. Interestingly, this process appears to be required for DSB repair by HR, although this is likely a distinct requirement to the role played by ATM-dependent KAP1 phosphorylation in relaxing HC at DSB sites.

The HP1β isoform of HP1 is also known to undergo a dynamic modification during the DDR. Indeed, HP1β is phosphorylated at T51 by Casein Kinase 2 (CK2) in response to DSB formation, triggering its rapid mobilisation from H3K9me3 sites to which it is bound (via its chromodomain) in undamaged cells [3,50]. The release of HP1β from methylated histone tails enabled another chromodomain-containing protein, the Tip60 histone acetyltransferase, to bind and effect modification of nearby factors [50]. Since H3K9me3 is abundant within HC, and indeed represents an HC-marker, it is tempting to speculate that these events are relevant to the HC DDR; however, the precise manner by which this occurs has yet to be clarified. Of note, Tip60 is part of the NuA4 chromatin remodeling complex that also encompasses Trrap, Rvb1 and the p400 ATP-dependent chromatin remodeling enzyme. Loss of any of these factors impacts upon DSB repair potentially via a role in regulating DSB-inducible acetylation of H4 [51,52]. They appear to have a specific function in regulating HR-mediated DSB repair. However, the relevance of this to HC-DSB repair is also unclear.

## 7. pKAP-1 Foci Formation Promotes Dispersal of the ATP-Dependent Chromatin Remodeling Enzyme, CHD3, from DSB Sites

The studies discussed above suggest that ATM triggers localized HC relaxation to promote DSB repair. Interestingly, KAP-1 phosphorylation results in pan nuclear chromatin relaxation as monitored by sensitivity to micrococcal nuclease [38]. However, these methods are insensitive and necessitate a high level of DSB formation (following exposure to high doses of IR or to the DSB inducing agent, neocarzinostatin) and hence, it is likely that they represent the consequence of pan-nuclear pKAP-1 rather than localized relaxation at HC-DSBs. As discussed above, the latter is 53BP1-dependent while the former is largely 53BP1-independent [28]. However, the dependence of such chromatin relaxation on 53BP1 has not been examined formally. Thus, there is currently no direct evidence that pKAP-1 foci elicit specific, localized HC relaxation at DSB sites.

**Figure 2:**
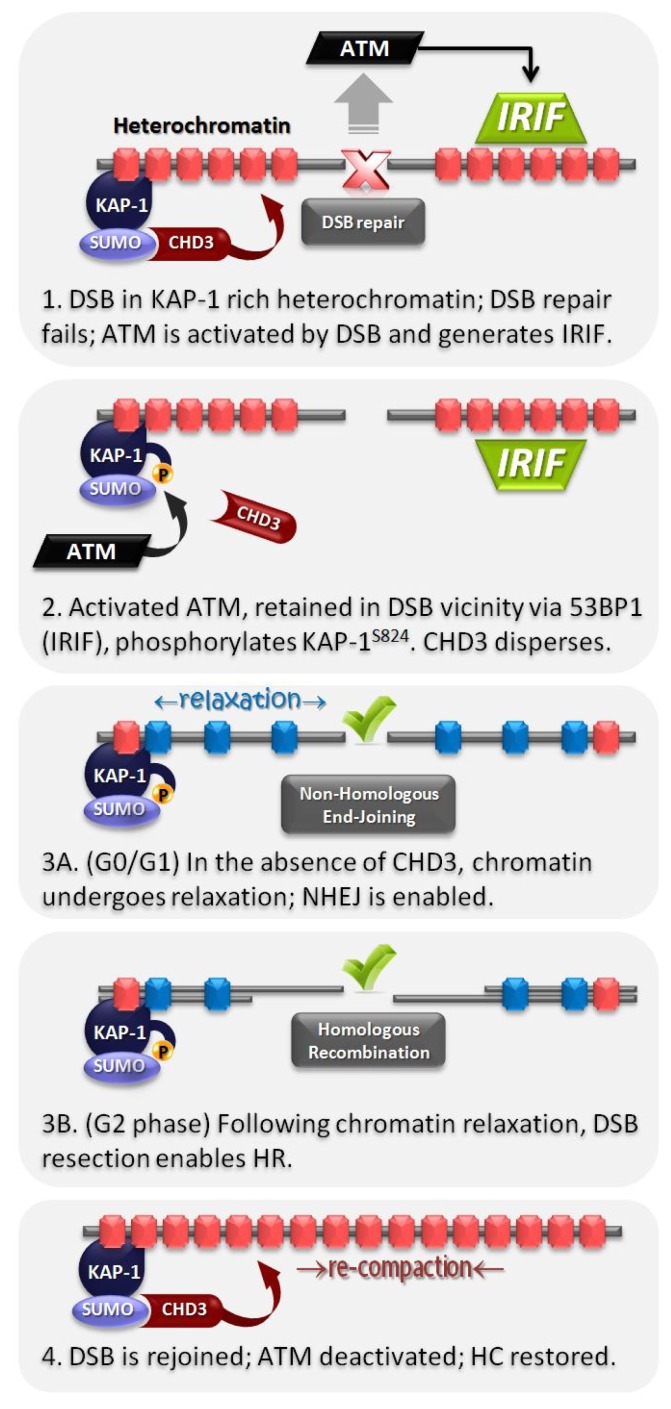
Ataxia telangiectasia mutated (ATM)-dependent heterochromatic DSB repair. (**1**) DSBs elicit ATM activation and IRIF formation, however repair processes within heterochromatin are inhibited by the compacted nucleosome configuration produced by KAP-1 dependent CHD3 activity; (**2**) Active ATM phosphorylates KAP-1 at S824, which interferes with the SUMOylation-dependent retention of CHD3 in chromatin; (**3**) In the absence of CHD3, the chromatin surrounding the DSB site relaxes, allowing (**3A**) non-homologous end-joining or (**3B**) if cells are in G2-phase, DNA end resection and homologous recombination mediated repair of the DSB; (**4**) Once the DSB is rejoined, ATM signaling is deactivated. Heterochromatic nucleosomes re-compact once KAP-1 is dephosphorylated and CHD3 activity is again retained.

Notwithstanding this limitation, there is compelling mechanistic evidence for how pKAP-1 foci formation leads to localized HC relaxation. The C-terminal region of KAP-1 encompasses several lysines that have been shown to undergo SUMOylation with SUMO1 (of which K554, K779, K804 are the most prominent), potentially via an auto-SUMOylation mechanism involving KAP-1’s *N*-terminal RING finger [53]. The S824 phosphorylation site of KAP-1 lies in a structurally-disordered region at the extreme *C*-terminal region. SUMOylation of KAP-1 is known to mediate interactions with the multi-subunit nucleosome remodeling and deacetylase (NuRD) complex as well as with the histone methyltransferase, SETDB1. The interaction between SUMOylated KAP-1 and NuRD occurs via the ATP-dependent chromatin remodeling subunit, CHD3 (specifically isoform 1, CHD3.1) [53,54]. Unlike the second isoform of CHD3 (CHD3.2), CHD3.1 contains a SUMO-interacting motif (SIM) at its extreme *C*-terminus, which, as its name implies, interacts with SUMO1-modified KAP-1. One possible model is that phosphorylation at S824 on KAP-1 might inhibit SUMOylation at the nearby residues, K804/K779/K554. However, although a previous finding was supportive of such a model [55], we were unable to observe any change in KAP-1 SUMOylation from 0.5 h to 8 h following 10–80 Gy IR. We did, however, observe a reduction in the level of chromatin bound CHD3 in the vicinity of the DSB following irradiation, which was ATM and 53BP1 (and hence pKAP-1)-dependent [56]. The model, supported by biochemical interaction studies, suggests that phosphorylation at the disordered *C*-terminal region of KAP-1 produces a SIM-like domain within KAP-1, which can interfere with the KAP-1^SUMO1^:CHD3^SIM^ interaction and causes CHD3 dispersal from the DSB site (Figure 2). This exquisitely dynamic mechanism is highly suited to confer rapid, reversible and highly localized HC relaxation in the vicinity of HC-DSBs. However, it is currently unclear whether the dispersal of CHD3 chromatin remodeling activity from HC-DSBs is sufficient to enable DSB-induced, ATM-dependent chromatin relaxation or whether additional factors are required.

## 8. Impact of HC on the Efficacy of Checkpoint Arrest

As mentioned above, DSBs that form within HC rapidly become localized to the HC periphery. Although IRIF expansion occurs slightly slower at these DSBs, at later times post IR their size is similar to those at EC regions, largely due to the expansion of γH2AX foci into the surrounding EC [27]. However, as discussed above, loss of HC compaction, either in human disorders with disordered HC or following knockdown of HC components such as KAP-1 by siRNA, results in larger γH2AX foci predominantly due to the spreading of γH2AX into the HC structure and a reduced requirement for ATM for HC-DSB repair [27,33]. The next question arising is whether the larger γH2AX foci derived in this situation confer any biological impact.

**Figure 3:**
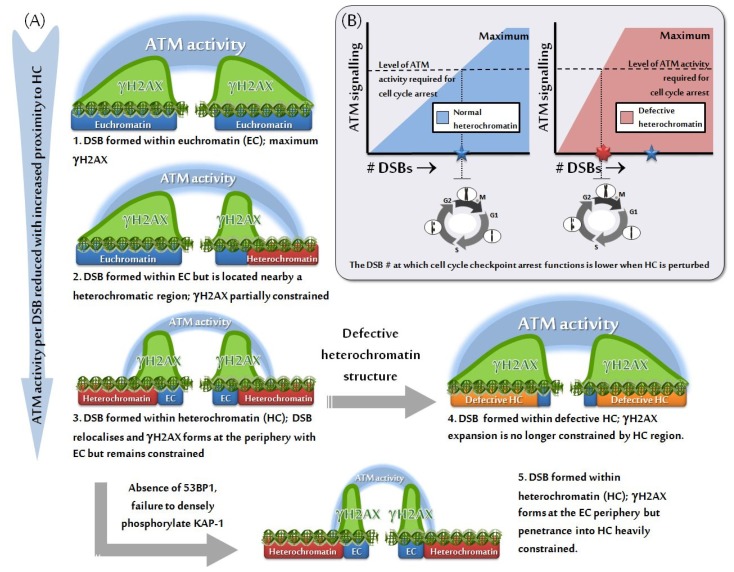
The influence of heterochromatin on ATM signaling and cell cycle checkpoint arrest. (**A**, 1–3) At early times after DSB formation, a significant difference in the magnitude of IRIF formation and consequential ATM signaling is observed based on the relative proximity of the DSB to heterochromatic centers, with the expansion of IRIF forming at DSBs located near or within heterochromatin BEING constrained. (**A**, 4) Where defective heterochromatin is encountered, as a result of germ-line mutation or siRNA-mediated knockdown of heterochromatic building factors, IRIF expansion is unconstrained and a greater degree of ATM signaling is produced relative to normal. (**A**, 5) In the absence of 53BP1 or the dense, localized phosphorylation of KAP-1, IRIF penetrance into heterochromatin is severely hindered causing a reduction in the amount of ATM signaling. (**B**) Cell cycle checkpoint arrest is triggered by a threshold number of DSBs in normal cells, due to a defined amount of ATM signaling being produced per DSB within the cell. The magnitude of ATM signaling per DSB is “set” by the natural balance between euchromatin and heterochromatin. Where defective heterochromatin is present, DSBs within or bordering on heterochromatic centers signal to a greater extent than they would in normal cells, since IRIF expansion and ATM signaling are no longer constrained. Hence, the number of DSBs required to achieve the minimum level of ATM signaling needed to trigger checkpoint arrest is lowered and these cells display hypersensitive checkpoint initiation and prolonged maintenance. Note that the figures display events at early times post irradiation. At later times, IRIF expansion occurs into euchromatin efficiently so that the overall size of the foci from DSBs within euchromatin versus heterochromatin are similar although most of the IRIF occurs within the euchromatin region. At later times, IRIF at DSBs within defective heterochromatin are larger than those within euchromatin suggesting that factors may be recruited to euchromatin to restrict their expansion.

Cell cycle checkpoint arrest represents a significant consequence of the DDR. Such checkpoints include the G1/S, intra-S and G2/M checkpoint. The G2/M checkpoint is interesting in this context since a range of studies have shown that low doses of ionizing radiation fail to efficiently activate checkpoint arrest—*i.e.*, there appears to be a defined threshold of DDR signaling required to activate checkpoint arrest [57,58]. Similarly, checkpoint arrest is not maintained until the completion of DSB repair. These findings prompted us to ask if loss of HC factors in human disorders or siRNA-mediated depletion of factors required to sustain the HC superstructure might impact upon the sensitivity of G2/M checkpoint arrest. The striking finding was that G2/M checkpoint arrest was activated following exposure to lower doses in Rett Syndrome cells compared to control cells and arrest was maintained for a prolonged period of time. Since DSB repair was normal in Rett Syndrome cells, this suggests that checkpoint arrest was activated and maintained by lower DSB numbers compared to control cell lines (depicted in Figure 3) [33]. A similar finding was observed with ICFa syndrome cells. Significantly, ICFa syndrome is characterized by premature ageing raising the possibility that hyper-activation of senescence could arise from enhanced signaling from uncapped telomeres.

## 9. Summary and Future Perspectives

Analysis of the DNA damage response in the past few years has revealed, somewhat unexpectedly, that the chromatin undergoes enormous changes following exposure to DNA damaging agents. The majority of these changes arise at the DSB site but pan-nuclear chromatin alterations have also been described. Perhaps surprisingly, these changes appear to effect both relaxation and compaction of chromatin, depending on the situation or time post damage. In this review, we have focused on those changes that take place when a DSB arises in HC-DNA. Although ATM signaling, which is known to exert a massive impact on chromatin in the DSB vicinity, appears to lie at the heart of the changes observed, some responses appear to be ATM-independent. However, critically, ATM is required for the repair of HC-DSBs. Further, a diminution of HC compaction, as observed in human disorders with HC-relaxation, leads to hypersensitive signaling and checkpoint arrest. Despite this, there remains only limited direct evidence that HC relaxation occurs at the level of a single DSB. One reason for this is that the magnitude of relaxation may be modest, either due to the nature of the relaxation or its extent, rendering it difficult to observe except by sophisticated and sensitive methods. Further innovative approaches are required to determine how the magnitude of HC compaction is modified at HC-DSBs. In this article, we have divided chromatin simply into “EC” or “HC”; however, chromatin is in fact composed of a highly diverse mosaic of chromatin states, with many shades of grey between the classic definition of transcriptionally active, open EC and compacted, silent HC. Specific questions that we feel need to be addressed are understanding the finer details of how chromatin compaction and specific epigenetic DNA and histone modifications combine to regulate DNA end resection and the outcome of repair, the magnitude of DDR signaling and its impact of cell cycle checkpoints. If HC DSBs more readily undergo DNA end resection, then it is important to determine its cellular consequence and whether HC affects the fidelity of the repair process and the likelihood of translocations.
